# The Met receptor tyrosine kinase prevents zebrafish primary motoneurons from expressing an incorrect neurotransmitter

**DOI:** 10.1186/1749-8104-3-18

**Published:** 2008-07-29

**Authors:** Alexandra Tallafuss, Judith S Eisen

**Affiliations:** 1 Institute of Neuroscience, University of Oregon, Eugene, OR, 97403, USA

## Abstract

**Background:**

Expression of correct neurotransmitters is crucial for normal nervous system function. How neurotransmitter expression is regulated is not well-understood; however, previous studies provide evidence that both environmental signals and intrinsic differentiation programs are involved. One environmental signal known to regulate neurotransmitter expression in vertebrate motoneurons is Hepatocyte growth factor, which acts through the Met receptor tyrosine kinase and also affects other aspects of motoneuron differentiation, including axonal extension. Here we test the role of Met in development of motoneurons in embryonic zebrafish.

**Results:**

We found that *met *is expressed in all early developing, individually identified primary motoneurons and in at least some later developing secondary motoneurons. We used morpholino antisense oligonucleotides to knock down Met function and found that Met has distinct roles in primary and secondary motoneurons. Most secondary motoneurons were absent from *met *morpholino-injected embryos, suggesting that Met is required for their formation. We used chemical inhibitors to test several downstream pathways activated by Met and found that secondary motoneuron development may depend on the p38 and/or Akt pathways. In contrast, primary motoneurons were present in *met *morpholino-injected embryos. However, a significant fraction of them had truncated axons. Surprisingly, some CaPs in *met *morpholino antisense oligonucleotide (MO)-injected embryos developed a hybrid morphology in which they had both a peripheral axon innervating muscle and an interneuron-like axon within the spinal cord. In addition, in *met *MO-injected embryos primary motoneurons co-expressed mRNA encoding Choline acetyltransferase, the synthetic enzyme for their normal neurotransmitter, acetylcholine, and mRNA encoding Glutamate decarboxylase 1, the synthetic enzyme for GABA, a neurotransmitter never normally found in these motoneurons, but found in several types of interneurons. Our inhibitor studies suggest that Met function in primary motoneurons may be mediated through the MEK1/2 pathway.

**Conclusion:**

We provide evidence that Met is necessary for normal development of zebrafish primary and secondary motoneurons. Despite their many similarities, our results show that these two motoneuron subtypes have different requirements for Met function during development, and raise the possibility that Met may act through different intracellular signaling cascades in primary and secondary motoneurons. Surprisingly, although *met *is not expressed in primary motoneurons until many hours after they have extended axons to and innervated their muscle targets, Met knockdown causes some of these cells to develop a hybrid phenotype in which they co-expressed motoneuron and interneuron neurotransmitters and have both peripheral and central axons.

## Background

Although different subtypes of motoneurons of invertebrate species use different neurotransmitters to activate muscle [1,2], all vertebrate motoneurons activate muscle via release of acetylcholine (ACh) [3]. Historically vertebrate motoneurons have been considered exclusively cholinergic. However, several recent studies provide evidence that mammalian spinal motoneurons release both ACh and glutamate from collaterals within the spinal cord that synapse with inhibitory interneurons known as Renshaw cells, although ACh is still thought to be the only neurotransmitter that mediates motoneuron activation of skeletal muscle [4-6]. It is unknown how two distinct neurotransmitters are differentially regulated within these motoneurons. But the importance of appropriate regulation is underscored by a recent study showing that forced expression of neurotransmitters other than ACh in frog motoneurons causes inappropriate expression of non-cholinergic receptors at the neuromuscular junction [7].

Expression of the correct neurotransmitter is crucial for normal nervous system function, although the mechanisms that establish appropriate neurotransmitter expression are not well understood. Interneurons in the chick spinal cord can be induced to express ACh inappropriately by forced expression of MNR2, Lhx3, or Islet1 transcription factors [8,9]. However, forced expression of these transcription factors causes the interneurons to initiate a program of motoneuron differentiation [8,9] for which ACh is the appropriate neurotransmitter, suggesting that neurotransmitter expression is established by programs that specify cell fate. On the other hand, it is well-known that at least some neural crest-derived neurons of the peripheral nervous system normally change their neurotransmitter phenotypes during development, and that this is regulated by environmental signals [10,11]. These studies show that under some conditions, neurotransmitter expression is altered in response to the environment after cell fate is specified. Consistent with this idea, changing calcium-mediated neural activity can regulate neurotransmitter expression in neurons in culture [12] and in vivo [7] without affecting expression of markers of cell fate specification [7]. Together these studies suggest that regulation of neurotransmitter phenotype is complex and involves both intrinsic factors that regulate differentiation programs as well as responses to environmental signals.

One environmental signal known to affect neurotransmitter phenotype in motoneurons is Hepatocyte growth factor (HGF; also known as Scatter factor). Axotomy of adult hypoglossal motoneurons leads to a dramatic loss of mRNA and protein of the ACh synthetic enzyme, choline acetyltransferase (ChAT); this loss can be prevented by administration of HGF [13]. HGF has also been shown to stimulate choline acetyltransferase activity in motoneurons *in vitro *[14]. HGF acts through the Met receptor tyrosine kinase [15], which is expressed in motoneurons and has been shown to be important for their development. For example, HGF acts through Met as an axonal attractant and survival factor for some populations of mammalian and avian motoneurons [14,16-22] and has also been shown to be required to recruit a subpopulation of motoneurons to a specific motor pool [23].

**Figure 1:**
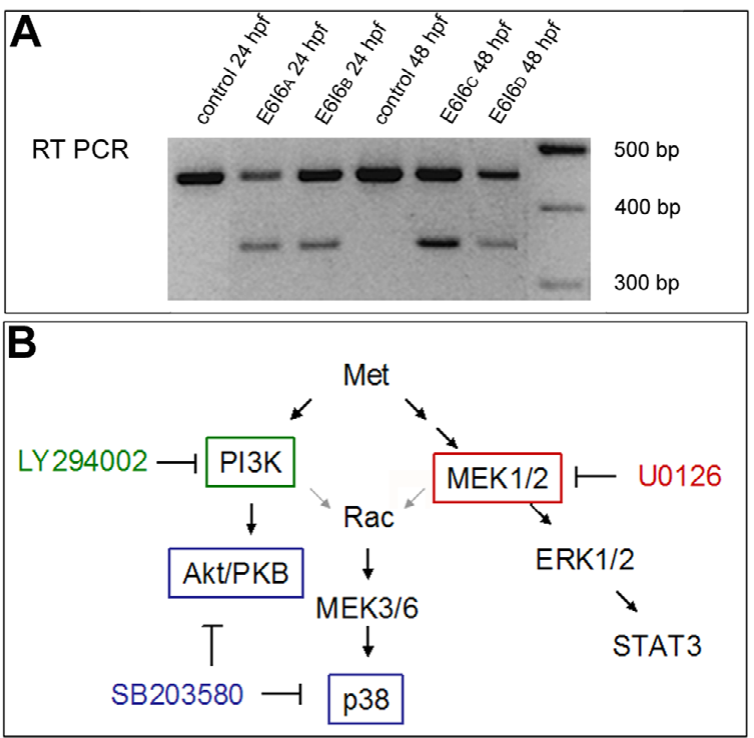
**Blocking Met function**. **(a) **RT-PCR showing that the E6I6 MO blocks *met *mRNA splicing. **(b) **Activation of Met can initiate intracellular signaling through several different downstream cascades, including MEK1/2 and PI3K [24,25]. This diagram shows the cascade and where specific inhibitors act. See text for details.

Experiments carried out using a variety of cell types have shown that activation of Met can initiate intracellular signaling through several different downstream cascades, including mitogen activated protein kinase (MAPK), phosphatidylinositol 3-kinase (PI3K) and p38 and Akt pathways [24,25]. These cascades can act independently or can be stimulated simultaneously, and there can be crosstalk among them [26-28]. In addition to activation via Met, these intracellular signaling pathways can also be activated by other receptors [29-33]. To elucidate the roles of these pathways in cellular events, a number of specific pathway inhibitory reagents have been developed, including LY294002, which inhibits the PI3K pathway [34], U0126, which inhibits the MAPK pathway by inhibiting MEK1/2 [35], and SB203580, which inhibits Akt [36] and p38 MAP kinase [37] (Figure 1). It is currently unknown which of these signaling cascades is activated by HGF-mediated Met signaling in motoneurons, and whether different cascades affect different aspects of HGF-mediated Met function in these cells.

In the present study, we have taken advantage of the experimental tractability of embryonic zebrafish to investigate the role of Met in motoneuron differentiation. Development of zebrafish spinal motoneurons has been well-characterized [38]. Zebrafish have two waves of motoneuron differentiation: primary and secondary motoneurons. Primary motoneurons (PMNs) constitute a small set of segmentally reiterated cells generated during gastrulation [38,39]. Each PMN is individually identified based on its morphology and gene expression pattern. Within each spinal hemisegment, CaP has the most caudally located cell body, RoP has the most rostrally located cell body, and MiP has a cell body located between CaP and RoP. Some spinal hemisegments have an additional PMN, called VaP, which is essentially a duplicated CaP that typically dies [40]. PMN axons pioneer nerve pathways followed later by axons of secondary motoneurons (SMNs) [41,42]. SMNs are born later than PMNs and are more numerous [38]. SMNs are born [43] and extend axons [42] over a protracted period of development; several studies suggest that there are distinct subsets of SMNs [44-47], although this has not yet been studied in detail. In addition to extensive characterization of their development, many recent studies have characterized the physiological properties of zebrafish motoneurons and how motoneurons are driven by interneurons to activate various types of behavior (see [38]). The neurotransmitters of zebrafish spinal interneurons have been extensively characterized. Specific subsets of interneurons have been shown to be glycinergic or glutamatergic [48,49]. In addition, several types of interneurons have been shown to express the neurotransmitter gamma-amino butyric acid (GABA) [48-50].

A previous study showed that zebrafish *met *is a specific marker of CaP and VaP [51]. We report here that a few hours later, *met *is also expressed in MiP and RoP, and that even later in development *met *is expressed in at least some, perhaps all, SMNs. We used morpholino antisense oligonucleotides (MOs) to knock down Met function and found that this had distinct effects on SMNs and PMNs. Many SMNs required Met for their differentiation and the SMN population was significantly reduced in *met *MO-injected embryos. In contrast, PMN differentiation appeared normal in *met *MO-injected embryos. However, some PMNs had truncated peripheral axons or developed interneuron-like processes within the spinal cord. In addition, in the absence of Met many PMNs inappropriately expressed GABA. Whether vertebrate motoneurons ever normally express GABA is controversial, and we return to this point in the discussion. To learn whether distinct Met-activated signaling cascades are responsible for the different phenotypes we observed following Met knock down, we used inhibitors that affect different pathways downstream of Met activation. Our results suggest that the p38 and/or Akt cascade may be required for SMN differentiation, whereas the MEK1/2 cascade may be required for appropriate neurotransmitter expression and to prevent formation of interneuron-like axons in PMNs. Together our results suggest that Met acts through different pathways to affect different aspects of motoneuron development.

## Materials and Methods

### Animal husbandry and lines

Zebrafish embryos were obtained from natural spawning of AB or AB/TU wild-type or *mn2Et *(also referred to as *parg*^*mn2Et*^) [52], *Tg(gata2:GFP) *[53] and *Tg(pax2a:GFP) *[54] transgenic lines. Fish were staged by hours post-fertilization at 28.5°C (hpf) [55].

### Cloning of zebrafish chat and met

We amplified two fragments, 1,400 bp and 900 bp from cDNA from a mixture of 24 hpf and 48 hpf embryos; the 1,400 bp fragment was amplified using primers CAT3 and CAT6 and the 900 bp fragment was amplified using primers CAT5 and CAT6. The fragments were cloned into TOPO-TA vector, sequenced and tested for specific expression patterns. Primer sequences were:

CAT3, 5'-ACAGGTTAGCACTACCTGTC-3';

CAT5, 5'-CTGAATGACAGCAACAGACG-3';

CAT6: 5'-TGGTCCGTCTGAGGATTGTAG-3'.

We cloned a 1.1 kb fragment of zebrafish *met *from cDNA from a mixture of 24 hpf and 48 hpf embryos using primers zfmetE1-1 and zfmetE1-2. The fragment was cloned into PCRII-TOPO and verified by sequence and expression pattern. Primer sequences were:

zfmetE1-1, 5'-ATGTGAGGAACCAATAGAAAGC-3';

zfmetE1-2, 5'-CAGATCCTGGAAAGTGACGG-3'.

### Downregulation of Met

To knock down Met activity, we used three MOs (GeneTools; Philomath, OR, USA): CM1a and CM2 were designed to block *met *(ENSDART00000104456; NCBI Entrez GeneID 492292) translation; these are the same MOs used by Haines and colleagues [56], thus, we repeated their experiments, showing the absence or reduction of *myoD *RNA expression in fin myoblasts in MO-injected embryos at 48 hpf, to verify that these MOs worked. CM1a was designed to anneal to ATG-containing sequences and CM2 was targeted against the 5' untranslated region of *met*. We also used an additional MO, E6I6, which was designed to block *met *mRNA splicing, leading to a deletion of exon 6 and a truncation of the Met protein. We determined that the E6I6 MO blocked *met *mRNA splicing by RT-PCR (Figure 1a). As a control, we used an MO similar to CM1a, but containing a 5 bp mismatch (CM1a-5 mm). About 2–4 nl of MO, diluted in water, were injected into the cytoplasm of one-cell-stage embryos. For most experiments, CM1a and CM2 were injected together, at concentrations at which each MO alone had no effect (CM1a, 0.6 mM; CM2, 0.8 mM); throughout this paper, embryos injected with a combination of CM1a and CM2 are referred to as *met *MO-injected embryos. Injection of E6I6 resulted in essentially the same phenotypes as injection of CM1a, CM2 or a mixture of the two translation blockers. MO sequences and concentrations were:

CM1a, 5'-ATAGTGAATTGTCATCTTTGTTCCT-3', 0.7–1.0 mM;

CM2, 5'-CTGTAAAATAAAGACACCTGTCGGA-3', 0.9–1.2 mM;

E6I6, 5'-GATTTGTGATGACTCTTACCACAAA-3', 0.7–1.0 mM;

CM1a-5 mm, 5'-ATACTCAATTCTCATGTTTCTTCCT-3', 1.0 mM;

underlines represent mismatches.

In some experiments we co-injected mouse *Met *mRNA (*mMet *in pSP64; generous gift of G. Vande Woude, Van Andel Research Institute, Grand Rapids, MI, USA) [15] together with the MOs. As a further control to test whether RNA diluted the MO to a non-effective concentration, we performed a set of control experiments in which we injected a similar amount of *lacZ *mRNA, *mMet *RNA, together with the MOs. Injection of *lacZ *mRNA alone did not affect neuronal development. In contrast, injection of *lacZ *mRNA plus MOs resulted in a phenotype similar to injection of MOs alone, showing that any effects of *mMet *RNA plus MO injections were specific to the *mMet *mRNA.

### Blocking Met downstream effectors with pharmacological inhibitors

Met activates a number of different signaling pathways [15,57,58] and inhibitors that block these pathways have been previously used to test Met function (see Figure 1b) [24]. We used the following inhibitors to test Met function in zebrafish: U0126 (InvivoGen; San Diego, CA, USA), which blocks MEK1 and MEK2 [35], LY294002 (InvivoGen), which blocks PI3K [34], and SB203580 (InvivoGen) which blocks p38 and Akt [36,37]. Because LY294002 had severe effects on overall development, we did not pursue its effects on motoneuron differentiation. For the inhibitor studies, embryos were dechorionated and incubated in embryo medium. Cell permeable inhibitor was added at 16 hpf; embryos remained in the inhibitor solution until further processed at either 26 hpf or 48 hpf. We performed dose-response experiments to determine the optimal inhibitor concentrations to use for experiments. We tested concentrations between 10 and 120 μM. In both cases we found that concentrations below 50 μM had no effect and concentrations above 80 μM were deleterious to embryonic development. Therefore, we used both U0126 and SB203580 at 60 μM.

### RNA *in situ *hybridization and immunohistochemistry

RNA *in situ *hybridization and immunohistochemistry were carried out according to standard protocols [59].

#### RNA *in situ *hybridization

The following antisense RNA probes were used: *islet2 *(*isl2*) [60], *glutamate decarboxylase 1 *(*gad1*, also known as *gad67 *[48]), *met *(1.1 kb fragment spanning 1–1,165 bp of NCBI sequence AY687384; Entrez GeneID: 492292) and *chat *(900 bp fragment spanning 1,099–1,967 of NCBI sequence XM682602; Entrez GeneID: 559274).

#### Immunohistochemistry

The following primary antibodies were used: JL-8 mouse anti-GFP (Chemicon; Temecula, CA, USA) was used at 1:200; zn1 (University of Oregon) was used at 1:150; znp1 (University of Oregon) was used at 1:750; rabbit anti-GABA (Sigma; St. Louis, MO, USA) was used at 1:1,000; anti-Alcam (Alcam was previously known as DM-GRASP, Neurolin, zn5 antigen, and zn8 antigen; University of Oregon) was used at 1:4,000; F59 [61] was used at 1:10; 4D9 [62] was used at 1:50. Primary antibodies were revealed using secondary antibodies coupled to Alexa Fluor568 (goat anti-rabbit 1:1,000; Invitrogen-Molecular Probes; Eugene, OR, USA); Alexa Fluor488 (goat anti-mouse 1:1,000; Invitrogen-Molecular Probes); Alexa Fluor546 (goat anti-mouse IgG1, 1:1,000; Invitrogen-Molecular Probes); Alexa Fluor488 (goat anti-mouse IgG2a, 1:1,000; Invitrogen-Molecular Probes); Alexa Fluor546 (goat anti-mouse IgG2b, 1:1,000; Invitrogen-Molecular Probes).

To reveal ACh receptor (AChR) clusters, embryos were fixed at 4°C for 4 h, rinsed in phosphate-buffered saline, incubated in 5 μg/ml α-bungarotoxin (αBTX-546; Invitrogen-Molecular Probes) in incubation buffer [phosphate buffered saline plus 0.1% Tween/20 (PBT) + 1% dimethyl sulfoxide (DMSO) + 5% normal goat serum (NGS)] for 30 minutes at room temperature, rinsed in PBT, and then followed by a regular immunohistochemistry protocol (adapted from [63] with minor modification).

Embryos were scored and photographed with a Zeiss Axioplan microscope and photographed using a Nikon Coolpix 995 digital camera or imaged using a Zeiss LSM5 confocal microscope.

### Acridine orange staining

For characterization of cell death, embryos were stained according to Williams and Holder [64], with minor modifications. Briefly, embryos were incubated for 20 minutes in 5 mg/ml Acridine Orange (Sigma) in embryo medium, washed three times for 5 minutes in embryo medium and observed under fluorescence microscopy with an fluoro-iso-thio-cyanate (FITC) filter.

### Behavioral assay

We used high-speed imaging to monitor trunk movements evoked by touch [63]. In our experiment, the head of the embryo was embedded in agarose in such a way that the trunk was not restricted in its movements. The embryo was stimulated with an insect pin mounted on a micromanipulator (Narishige; East Meadow, NY, USA). The stimulus was repeated 5 times at 1 s intervals and recorded using a high-speed digital video camera (Pixelink; Ottawa, ON, Canada) at 100 frames per second. Movies were analyzed using Quicktime (Apple) and individual frames were assembled for presentation in Adobe Photoshop.

## Results

### *met *is expressed in a subset of zebrafish spinal motoneurons

**Figure 2:**
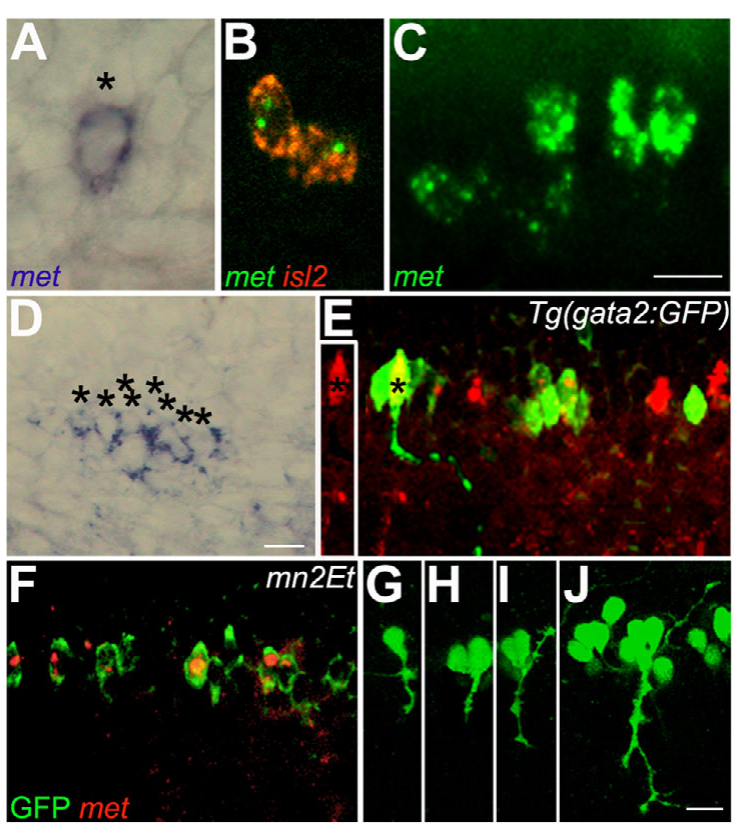
**Zebrafish *met *is expressed in developing spinal motoneurons**. All photographs are dorsal to the top and anterior to the left; this is also the case for subsequent figures except where noted. **(a) **A 22 hpf embryo showing *met *RNA expression in one cell of a spinal hemisegment (asterisk). **(b) **A 22 hpf embryo showing co-expression of *met *(green) and *islet2 *(red) in CaP and VaP. **(c) **A 26 hpf embryo showing *met *expression in all four PMNs in this spinal hemisegment. **(d) **A 48 hpf embryo showing *met *expression in eight cells (asterisks) in this hemisegment. **(e) **A 48 hpf *gata2:GFP *transgenic embryo showing GFP expression in ventrally projecting SMNs (green) and *met *expression (red) in a subset of these cells. The axon of the SMN labeled with an asterisk is shown as it projects out of the spinal cord toward its ventral muscle target. The inset to the left shows the same SMN, also marked with an asterisk, in only the red channel, clearly revealing that the SMN expresses *met *RNA. **(f) **A 48 hpf *mn2Et *transgenic embryo showing GFP expression (green) in PMNs and SMNs; *met *(red) is expressed in a subset of these cells. **(g-j) ***mn2Et *transgenic embryos at 24 hpf showing GFP expression in motoneurons: posterior segment showing GFP expression in CaP (g); posterior segment showing GFP expression in CaP and VaP (h); a more anterior segment showing GFP expression in CaP and MiP (i); an even more anterior segment showing GFP expression in CaP, VaP, MiP, RoP and several SMNs (j). Note that even as late as 5 days post-fertilization, in *mn2Et *embryos GFP-positive cells in the spinal cord all appear to have peripheral axons and no interneuron-like cells express GFP, suggesting that in the *mn2Et *line GFP is expressed exclusively in motoneurons. Scale bars, 10 μm.

*met *expression began in 1–2 cells in the ventral region of each spinal hemisegment at 22 hpf (Figure 2a), but within a few hours it was expressed in many more cells (Figure 2c). To learn which cells were *met*-positive, we first asked whether *met *is co-expressed with *islet2*, a specific marker of CaP and VaP at 22 hpf [60]. We found, as previously reported [51] that at this stage *met *was specifically expressed in CaP and VaP (Figure 2b), but not in other PMNs. However, by 26 hpf, *met *was expressed in all PMNs (Figure 2c). Later, by 48 hpf, *met *was detectable in clusters of four to eight cells in the ventral region of each spinal hemisegment (Figure 2d). To determine whether these *met*-positive cells were SMNs, we used transgenic embryos that express green fluorescent protein (GFP) under the control of the *gata2 *promoter [*Tg(gata2:GFP)*], which has been shown to be expressed in ventrally-projecting SMNs [52] (Figure 2e) and in some interneurons [53], and is routinely used to study SMN development [65,47,46,68]. We also used embryos of the *mn2Et *line that express GFP under the control of the *parg *promoter [52] (Figure 2f). GFP has been previously reported to be expressed specifically in CaP in the *mn2Et *line [52]. However, we find that, in this line, GFP is expressed in all zebrafish PMNs (Figure 2g–j), and in at least some zebrafish SMNs (Figure 2j). At 48 hpf, in *mn2Et *embryos all *met*-positive cells co-expressed GFP, but not all GFP-positive cells expressed *met *(Figure 2f) suggesting that *met *is expressed in at least some SMNs. We also found that many, but not all, *met*-positive cells co-expressed *gata2*:*GFP *(Figure 2e); cells expressing *met *mRNA but not GFP in *Tg(gata2:GFP) *embryos are probably PMNs and dorsally projecting SMNs that are *gata2:GFP*-negative. Together these results provide evidence that *met *is initially expressed only in CaPs and VaPs, but soon after it is expressed in other PMNs and later it is expressed in SMNs. These results do not allow us to conclude whether or not *met *is ever expressed by dorsally-projecting SMNs. In addition, because SMNs are born over a protracted period of development [43], we also cannot conclusively determine whether *met *is expressed by all ventrally-projecting SMNs or only by a subset of these cells.

### Met is required for normal touch-evoked behavior

Expression of *met *in PMNs and at least some SMNs prompted us to ask whether Met function is necessary for proper regulation of motoneuron-mediated behaviors. Zebrafish embryos begin to exhibit spontaneous muscle contractions that result in coiling movements shortly after PMN axons first extend out of the spinal cord [69-71]; spontaneous coiling requires functional PMNs [71]. *met *MO-injected embryos had normal spontaneous coiling, suggesting that some PMNs were present and functional.

**Figure 3:**
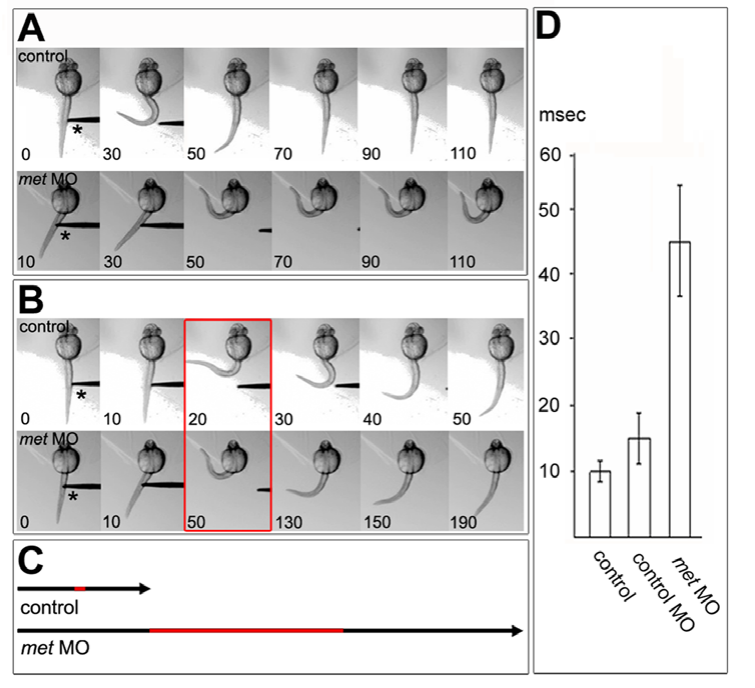
**Normal touch-evoked movements require Met function**. Embryos are oriented anterior to the top and viewed from dorsal. **(a) **Control embryo (top panel), head embedded in agarose, responded to touch by bending away from the probe (asterisk); times indicated are milliseconds. A *met *MO-injected embryo (bottom panel) responded to touch significantly more slowly than the control. Data from the control and *met *MO-injected embryo are shown on the same time scale. **(b) **The same embryos as in (a), but for each embryo the entire time-course of the movement is shown. The entire touch response took about 50 ms in the control embryo, but took about 190 ms in the MO-injected embryo, which remained in the coiled position for about 60 ms. Red box indicates maximal bending. **(c) **Black arrows display the time span of one touch response, as shown in (b), for control and *met *MO-injected embryos; the red section indicates the time the embryo stayed in a coiled position. **(d) **Time frame in which control and injected embryos stayed in the coiled position [red in (b,c)]. The differences between control and control MO-injected embryos were not significantly different, but they were both significantly different from *met *MO-injected embryos (*p *< 2.6 × 10^-13^, n = 46 touch-evoked responses of 8 control embryos and 32 touch-evoked responses of 8 *met *MO-injected embryos; *p *< 1.07 × 10^-7^, n = 22 touch-evoked responses of 4 control MO-injected embryos and 32 touch-evoked responses of 8 *met *MO-injected embryos.

At later stages of development, embryos stop coiling spontaneously and instead respond to touch on the head or tail [71]. Therefore, we compared motility of control and *met *MO-injected embryos at 28–30 hpf, a stage at which control embryos are touch-responsive but no longer coil spontaneously. Control embryos responded to tail touch with a stereotyped bend of the trunk (see [70]); the average time for completing the stereotyped movement was about 50 ms (Figure 3a,b). *met *MO-injected embryos had impaired tail touch-evoked motility (Figure 3a,b). They moved slower, with an average time for completing the movement of 190 ms, and often had spasmodic movements instead of the smooth bending seen in controls. The time-course of the touch-evoked behavioral response was significantly different between *met *MO-injected and control embryos, but was not significantly different between *met *mismatch MO-injected and control embryos (Figure 3d), showing that normal Met function is required for proper tail touch-evoked movements.

**Figure 4:**
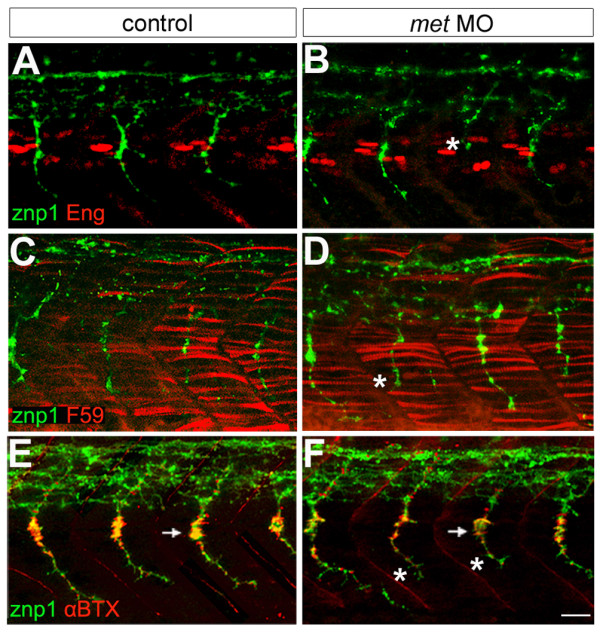
**Met appears unnecessary for muscle and neuromuscular junction formation**. **(a,b) **Engrailed antibody (Eng, red) labeling showing muscle pioneer cells and znp1 antibody labeling showing motor axons (green). In *met *MO-injected embryos, some CaP axons are truncated (asterisk). **(c,d) **F59 antibody (red) labeling showing fast muscle fibers and znp1 antibody staining showing motor axons (green). F59 labeling appears the same in control (c) and *met *MO-injected (d) embryos, which have some truncated CaPs (asterisk). **(e-f) **αBTX (red) labeling showing AChRs and znp1 antibody labeling showing motor axons (green). The distribution of AChRs appears the same in control (e) and *met *MO-injected embryos (f) that have some truncated CaP axons (asterisks); however, it appears that the number of AChRs may be decreased at the myoseptal varicosity (arrows) by MO injection. For each experiment, 8 spinal hemisegments plus somites were examined in each of 21–33 *met *MO-injected embryos and 8 spinal hemisegments plus somites in each of 15 controls. Scale bar, 20 μm.

Even though *met *MO-injected embryos responded significantly slower to tail touches than did control embryos, their ability to move suggested that muscle function was normal and that neuromuscular junctions were present. We verified this by labeling 26 hpf control and *met *MO-injected embryos with zn1 or znp1 antibodies that recognize zebrafish PMNs [69,72] and antibodies that recognize specific muscle cell types, including anti-Engrailed that recognizes a specific subset of slow muscle fibers [62,73] (Figure 4a,b), and F59, that recognizes fast muscle fibers (Figure 4c,d) [74]. PMNs were present in *met *MO-injected embryos, although in some cases CaP axons were truncated (see below). Engrailed and F59 labeling were both present in *met *MO-injected embryos and appeared similar to controls. We also examined the localization of AChR clusters using αBTX [75]. AChR clusters were present in *met *MO-injected embryos and had a similar distribution as in controls (Figure 4e,f). Together these observations raise the possibility that Met function is not required for formation of muscles or neuromuscular junctions and that the impaired tail touch-evoked motility of *met *MO-injected embryos resulted from a requirement for Met for normal differentiation of PMNs, SMNs or both.

### Met plays a role in formation of ventral motor nerves

**Figure 5:**
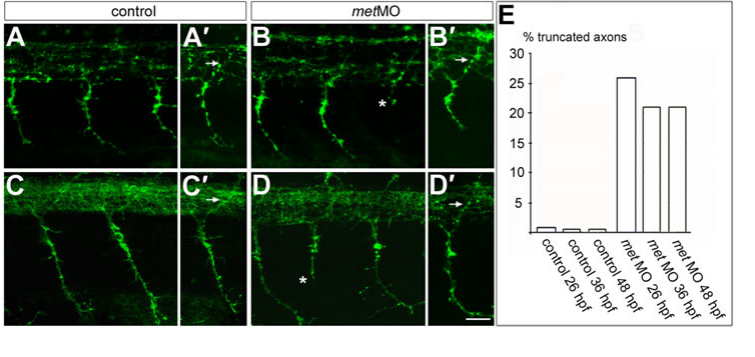
**Met is required for normal CaP axons. **(a-b') **Embryos at 26 hpf showing PMN axons labeled with znp1 antibody**. *met *MO-injected embryos have normal MiP axons [arrow in (b')] compared to controls [arrow in (a')]. However, some CaP axons are truncated in *met *MO-injected embryos [asterisk in (b)] compared to controls (a). **(c-d') **Embryos at 48 hpf showing PMN and SMN axons labeled with znp1 antibody. As at 26 hpf, dorsally projecting axons in *met *MO-injected embryos appear normal [arrow in (d')] compared to controls [arrow in (c')]. However, some ventrally projecting axons are truncated in *met *MO-injected embryos [asterisk in (d)], compared to controls (c). **(e) **Average percentage of truncated axons in control and *met *MO-injected embryos at 26, 36 and 48 hpf. n (26 hpf) = 8 somites in each of 12 embryos; n (36 hpf) = 8 somites in each of 10 embryos; n (48 hpf) = 8 somites in each of 11 embryos. Scale bar, 20 μm.

To learn whether Met is required for normal differentiation of PMNs and/or SMNs, we labeled 26 hpf and 48 hpf embryos with the znp1 antibody, which reveals primary and secondary motor axons [76]. By 26 hpf, PMN axons had extended both ventrally and dorsally in control embryos (Figure 5a,a'). In *met *MO-injected embryos, dorsally projecting MiP axons appeared normal, but some ventrally extending CaP axons were truncated (Figure 5b,b'). However, only about 25% of CaP axons were affected by Met knock down (Figure 5e).

By 48 hpf, SMNs had formed both dorsal and ventral motor nerves (Figure 5c–d'). In *met *MO-injected embryos the ventral nerves appeared thinner than in control embryos (Figure 5c,d). Because znp1 labels both PMNs and SMNs, and in 75% of hemisegments CaP axons had extended normally, this result suggests that at least some SMN ventral axons were truncated or failed to extend when Met was knocked down. In addition, there were some truncated ventral nerves. Previous studies showed that CaP is unnecessary for extension of SMN ventral axons [42]. Thus, the truncated ventral nerves could represent truncated CaP axons in segments in which SMN axons failed to extend. Alternatively, they could represent a combination of truncated CaP and truncated SMN axons. Together these observations suggest that Met is important for normal ventral axon extension by both PMNs and SMNs. We provide further tests of this hypothesis below.

### Normal secondary motoneuron differentiation requires Met signaling

**Figure 6:**
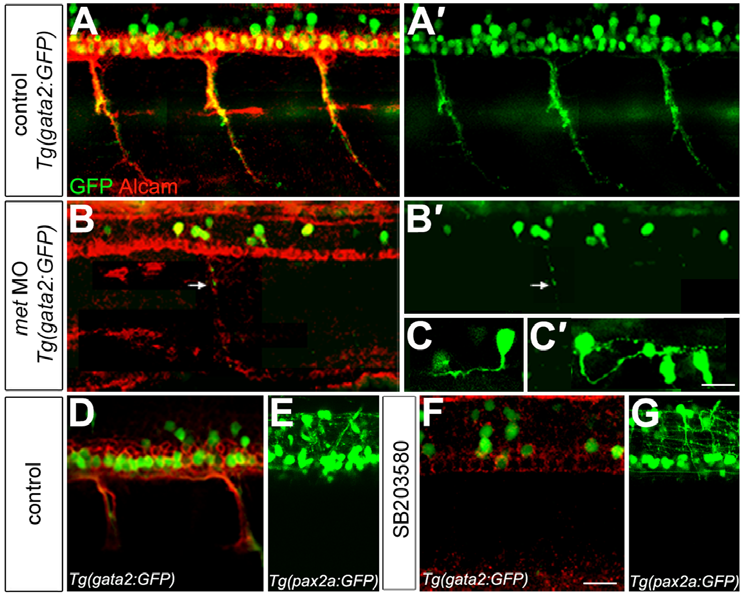
**Met is required for secondary motoneuron formation**. **(a-b') ***gata2:GFP *(green) transgenic embryos at 48 hpf labeled with antibody to Alcam (red). *gata2:GFP *is expressed in ventrally projecting SMNs, some of which also express Alcam (a); (a') shows only the GFP. *met *MO-injected embryos (b,b') show a severe decrease in SMNs, but Alcam labeling of the floor plate appears normal. A small number of SMNs form and make ventral projections (arrow). (b) Both markers shown; (b') only GFP shown. **(c,c')**. Some of the GFP-positive cells in *met *MO-injected embryos show interneuron-like axons. (c) An interneuron with an ascending axon; (c') an interneuron with a descending axon. The two GFP-positive cells to the right in (c') are SMNs. **(d-g) ***gata2:GFP *transgenic embryos (green) at 48 hpf labeled with Alcam antibody (red). Exposure to SB203580 (f) severely decreased the formation of SMNs relative to controls (d), but the overall architecture of the spinal cord appeared normal based on expression of GFP driven by the *pax2a *promoter (e,g). Scale bar, 20 μm in all panels except (c,c'), which is 10 μm.

To learn whether Met was required for normal SMN development, we examined SMNs in *met *MO-injected *Tg(gata2:GFP) *embryos at 48 hpf. We also labeled these embryos with an antibody to Alcam, a cell surface protein expressed on floor plate and transiently on the somata and fasciculated segments of SMN axons [77]. In 48 hpf *Tg(gata2:GFP) *embryos, most of the GFP-positive SMN somata were also Alcam-positive (Figure 6a,a'). However, there were many more Alcam-positive somata than GFP-expressing somata, consistent with the observation that *gata2*-driven GFP is expressed in ventrally projecting SMNs but not in dorsally-projecting SMNs [53,66,67]. In addition, *Tg(gata2:GFP) *embryos had some dorsally-located, GFP-expressing somata that were Alcam-negative (Figure 6a,a'). These cells could be SMNs that have down-regulated Alcam expression or, alternatively, they might be GFP-expressing interneurons [53]. In contrast to control *Tg(gata2:GFP) *embryos, in *met *MO-injected *Tg(gata2:GFP) *embryos the number of ventrally projecting SMN somata and axons was severely reduced (Figure 6b,b'; Table 1) and some of the GFP-positive somata projected interneuron-like axons within the spinal cord rather than peripheral axons (Figure 6c,c'; Table 1). These cells might be interneurons that normally express GFP in this transgenic line. Alternatively, they could be SMNs that have developed as interneurons. Consistent with the latter possibility, the number of GFP-positive somata just dorsal of the SMN soma domain (more than three cell diameters dorsal of the floor plate) was increased in *met *MO-injected embryos (Table 1). It is clear that in *met *MO-injected embryos there are several types of GFP-positive interneurons, because some of them have ascending axons (Figure 6c) whereas others have descending axons (Figure 6c'). To learn whether Met knock down caused SMNs to die, we labeled embryos with acridine orange to reveal dying cells [78] at 28, 36, and 48 hpf, but saw no significant difference between the spinal cords of *met *MO-injected embryos and controls (data not shown). In addition, the overall cellular structure of the spinal cord appeared entirely normal in *met *MO-injected embryos (data not shown). Together these results suggest that Met is required for formation of at least some SMNs and raise the possibility that when Met is knocked down, at least some SMNs develop as interneurons.

**Table 1:** The number of second motoneurons is reduced following Met knock down

	Control	*met *MO	SB203580
Number of embryos	18	20	16
Percent of segments with ventral nerve	98	61	39
GFP^+ ^somata within 3 cell diameters of floor plate	20 ± 0.47	9 ± 0.61	6 ± 0.40
GFP^+ ^somata 3 cell diameters dorsal of floor plate	5 ± 0.39	10 ± 0.52	8 ± 0.68

GFP-positive SMN axons (three segments/embryo) and somata (two segments/embryo) were analyzed in 48 hpf *Tg(gata2:GFP) *embryos. Soma counts of treatment groups are all significantly different from controls (*p *< 0.00015).

### Met may mediate secondary motoneuron development through activation of p38 and/or Akt

Activation of the Met receptor can initiate signaling through several different intracellular cascades (Figure 1) [15,57,58]. To investigate which of these downstream pathways is involved in SMN differentiation, we treated embryos with compounds designed to inhibit specific intracellular signaling cascades activated by Met and other receptors, and assayed SMN development at 48 hpf. Specifically, we used U0126, which has been shown to inhibit MEK1/2 (Figure 1) [35], and SB203580, which has been shown to inhibit Akt and p38 (Figure 1) [34,36]. The MEK1/2 inhibitor U0126 had no effect on SMN development (Table 2 and data not shown), indicating that the MEK1/2 pathway is not involved. In contrast, the Akt and p38 inhibitor SB203580 dramatically affected SMN development (Table 2). In fact, SMN development was even more severely affected by blocking p38 and Akt signaling than by knocking down Met function with MOs: inhibitor-treated embryos were missing essentially all SMN somata and axons (Figure 6d–g). There is some correctly spliced *met *mRNA following MO injection (Figure 1), suggesting that the more severe effect of the inhibitor could at least partially result from a more complete knock down of Met signaling than is achieved in *met *MO-injected embryos. This result also raises the possibility that in addition to Met, other pathways act upstream of p38 and/or Akt during SMN formation. Future experiments to learn the identities of these other pathways will help elucidate the mechanisms required for normal SMN development. In addition, zebrafish has two *p38 *genes, *p38a *and *p38b*, that are broadly expressed at the stages of development we have studied [79], raising the possibility that some effects on SMNs could be cell non-autonomous. Thus, to test whether SB203580 had a general effect on ventral spinal cord neurons, we treated *Tg(pax2a:GFP) *embryos [54] with SB203580 and examined their spinal cords. Spinal cord architecture appeared essentially normal in SB203580-treated *Tg(pax2a:GFP) *embryos (Figure 6d–g) suggesting that this inhibitor specifically affected SMNs. Together these results suggest that Met may act through the Akt and/or p38 cascades to promote formation of zebrafish SMNs.

**Table 2:** SB203580 affects SMN development

	Number of embryos	Percent of segments with reduced SMN somata
Control	75	0
*met *MO	96	80
U0126	20	0
SB203580	28	90

SMN somata were analyzed in three segments of each embryo at 48 hpf. In all cases, the three segments had the same phenotype; in other words, within an embryo all segments had reduced SMN somata or no segments had reduced SMN somata.

Surprisingly, although SB203580-treated embryos lacked most SMNs, at 30 hpf their tail touch-evoked motility was essentially indistinguishable from control embryos (data not shown). Only a few SMNs have projected axons by this stage [42]; thus, this result suggests that at this stage most of the touch response is mediated by activity of PMNs, rather than by activity of SMNs, although this has not yet been tested directly. If this is the case, then the movement defects we observed at 30 hpf in *met *MO-injected embryos would have to arise from a requirement for Met in PMN differentiation, rather than from the decrease in SMN number. To test whether this was the case, we examined the effects of Met knock down on PMN development.

### Met prevents CaPs from extending interneuron-like axons within the spinal cord

**Figure 7:**
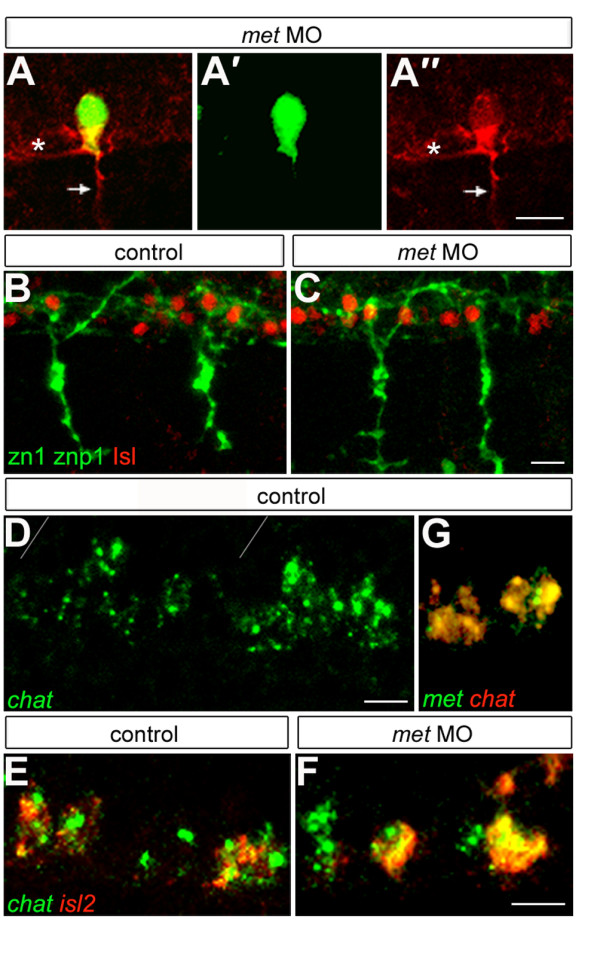
**Met is required for aspects of CaP identity**. **(a) **A *met *MO-injected *mn2Et *(green) transgenic embryo labeled with znp1 antibody (red) showing that CaP has both a peripheral axon (arrow) and a central axon (asterisk). **(a') **The same cell showing only GFP. **(a") **The same cell showing only znp1 labeling. **(b,c) **Islet1/2 antibody (Isl; red) and zn1 plus znp1 antibodies (green) labeling showing that CaPs in *met *MO-injected embryos (c) co-express these markers as they do in controls (b). For each condition we examined eight spinal hemisegments per embryo. n = 56 *met *MO-injected embryos and 32 control embryos. **(d) ***chat *is expressed in somata in the normal location of PMNs; gray lines show segment boundaries. **(e,f) **Expression of *chat *(green) and the CaP and VaP-specific marker *islet2 *(red) shows that in *met *MO-injected embryos (f) CaPs express *chat*, as they do in controls (e). For each condition we examined eight hemisegments per embryo. n = 24 *met *MO-injected embryos and 15 control embryos. **(g) **Co-expression of *chat *(red) and *met *(green) in PMNs. Scale bars, 10 μm.

Despite the motility defects in *met *MO-injected embryos, three quarters of the CaP axons looked essentially normal as judged by their morphology (Figure 5), suggesting that Met might be required for proper development of CaP characteristics other than the peripheral axon. To learn whether Met was required for differentiation of other features of CaP morphology, we examined CaPs in *met *MO-injected *mn2Et *embryos and we labeled embryos with zn1 and znp1 antibodies. Surprisingly, we found that many CaPs had an interneuron-like process within the spinal cord, in addition to the normal peripheral axon (Table 3; Figure 7a–a"). To learn whether there was a correlation between CaPs with truncated axons and those with interneuron-like axons, we analyzed morphology of a subset of CaPs (Table 4). There did not appear to be a correlation, as we found that both CaPs with normal-appearing peripheral axons and CaPs with truncated peripheral axons had interneuron-like axons. These results suggest that Met is important in maintaining CaP morphology by preventing CaP from forming an interneuron-like central process.

**Table 3:** Met knockdown results in CaPs with both peripheral and central axons

	Number of embryos	Number of CaPs with only a peripheral axon	Number of CaPs with peripheral and central axons
Control	48	139	5
*met *MO	56	139	29
U0126	45	117	18
SB203580	27	81	0

CaPs were analyzed in 26 hpf *mn2Et *embryos; 3 hemisegments were examined per embryo.

**Table 4:** Whether CaP has an interneuron-like axon appears uncorrelated with whether it has a truncated peripheral axon

	Control 23 CaP axons in 6 embryos		*met* MO 33 CaP axons in 10 embryos		U0126 55 CaP axons in 8 embryos	
			
Peripheral axon extended to	No interneuron- like axon	Interneuron- like axon	No interneuron- like axon	Interneuron- like axon	No interneuron- like axon	Interneuron- like axon
Ventral edge of muscle	22	1	17	8	36	10
Horizontal myoseptum	1	0	1	1	5	2
Just out of neural tube	0	0	4	2	2	0

Axons were analyzed in *mn2Et *embryos at 26 hpf.

### Met prevents CaPs from expressing an interneuron-specific neurotransmitter

Based on their morphology, many CaPs in *met *MO-injected embryos appeared to have a hybrid identity that combined features of both motoneurons and interneurons. To learn whether this hybrid identity extended to molecular features, we assayed two aspects of CaP: expression of Islet proteins and neurotransmitter phenotype. We found that CaPs in *met *MO-injected embryos had normal expression of Islet proteins (Figure 7b,c), suggesting that they retained motoneuron identity. To learn whether CaPs expressed normal cholinergic properties or expressed interneuron-specific neurotransmitters, we tried several antibodies to ChAT, the ACh synthetic enzyme, but none of them worked in our hands. Because the *chat *sequence is highly conserved within vertebrates, we used the goldfish *chat *sequence [80] to blast zebrafish databases (NCBI) and found a hypothetical sequence with high homology to all vertebrate *chat *sequences. We amplified *chat *from zebrafish cDNA, verified the sequence and found that it was expressed in cells with the correct position and morphology to be PMNs (Figure 7d). We confirmed *chat *expression in CaPs using double fluorescent *in situ *hybridization with *islet2 *(Figure 7e), and also confirmed that CaPs co-express *met *and *chat *(Figure 7g). We analyzed *chat *expression in CaPs in *met *MO-injected embryos at 24 hpf and found that it was unaltered relative to controls (Figure 7e,f), showing that this aspect of CaP identity did not depend on Met function.

**Figure 8:**
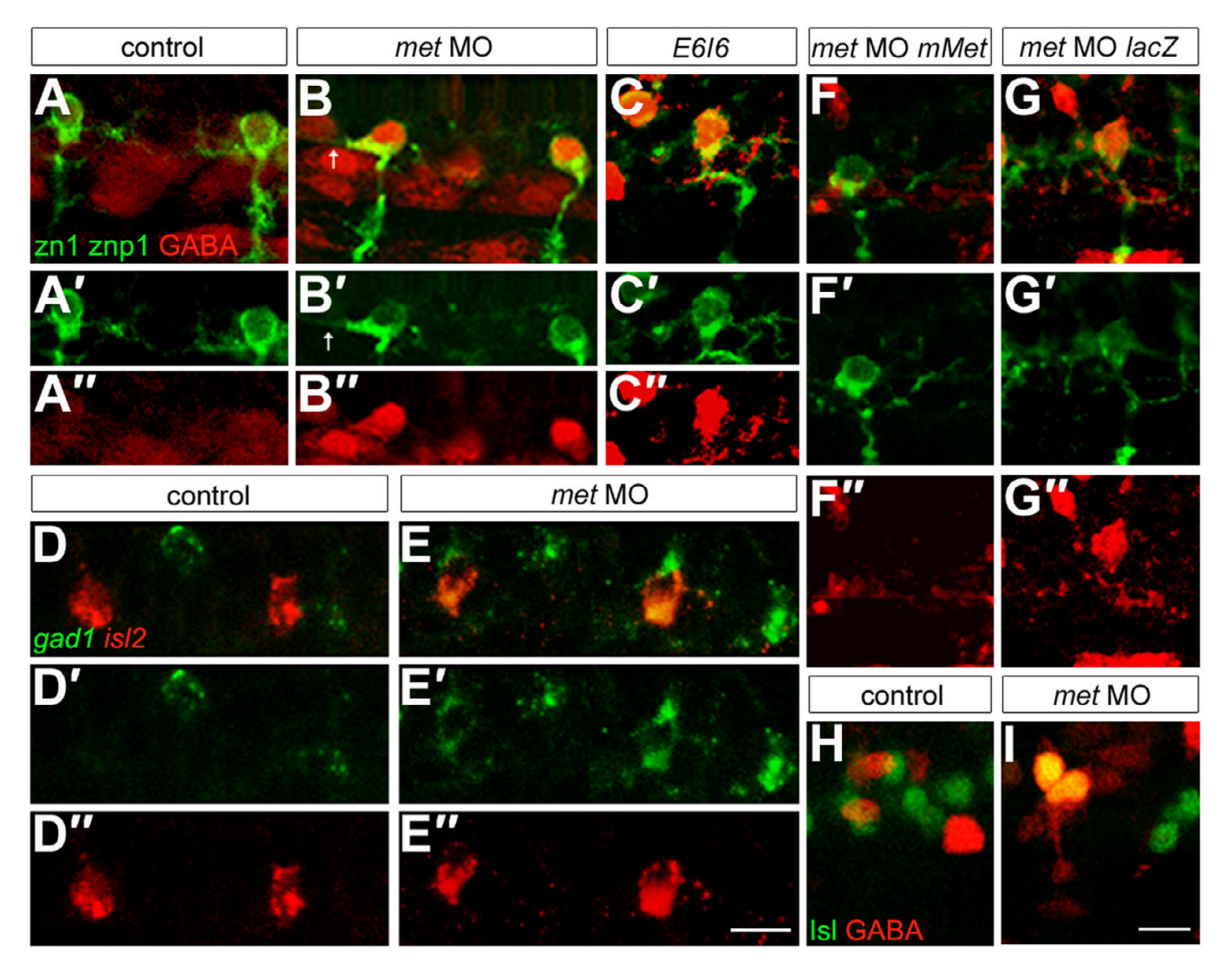
**Met is required to prevent CaPs from expressing an inappropriate neurotransmitter**. For each condition we examined eight spinal hemisegments per embryo. **(a-c") **Embryos at 26 hpf labeled with antibodies to GABA (red) and zn1 plus znp1 (green); (a',b',c') only the green channel is shown; (a",b",c") only the red channel of the micrographs shown in (a,b,c) is shown. CaPs expressed GABA in embryos injected with translation blocking (b-b") or splice blocking (c-c") *met *MOs, but not in controls (a-a"). Note that some CaPs in *met *MO-injected embryos also have ectopic axons within the spinal cord (arrows). **(d-e") **Embryos at 26 hpf labeled with riboprobes to *gad1 *(green) and *islet2 *(red). (d',e') Only the green channel is shown; (d",e") only the red channel of the micrographs shown in (d,e) is shown. In *met *MO-injected embryos, *islet2*-positive CaPs also express *gad1 *(e-e"), whereas these two genes are not co-expressed in CaPs of control embryos (d-d"). **(f-g") **Embryos at 26 hpf labeled with antibodies to GABA (red) and zn1 plus znp1 (green). (f',g') Only the green channel is shown; (f",g") only the red channel of the micrographs shown in (f,g) is shown. CaPs do not express GABA in embryos co-injected with *met *MO and *mMet *mRNA (f-f"), but do express GABA in embryos co-injected with *met *MO and *lacZ *mRNA (g-g"). **(h,i) **Embryos at 26 hpf labeled with antibodies to GABA (red) and Islet (green). CaPs co-express Islet and GABA in *met *MO-injected embryos (i) but not in controls (h). n = 80 embryos in (a); n = 128 embryos in (b); n = 24–32 embryos each for (c,e,f,g); n = 20 embryos for (h); n = 35 embryos for (i). The phenotype was seen in 70% of embryos injected with the splice-inhibitor MO and 80% of embryos injected with translation-blockers; in affected embryos all segments showed the phenotype. Rescue was seen in 90% of embryos injected with *mMet *mRNA and 0% of embryos injected with *lacZ *mRNA. Scale bars, 10 μm.

We previously showed that in the absence of Islet1, zebrafish PMNs develop interneuron-like axons rather than their normal peripheral projections and that some of these cells express the interneuron-specific neurotransmitter GABA [81]. Therefore, we asked whether CaPs expressed GABA when Met function is knocked down. We found that in embryos injected with either translation blocking or splice blocking *met *MOs, the majority of CaPs and VaPs expressed GABA (Figure 8a–c") in contrast to control embryos, in which no CaPs or VaPs expressed GABA (Table 5). We confirmed this by showing that in contrast to controls, in *met *MO-injected embryos CaPs and VaPs co-expressed GABA and Islet (Figure 8h,i). We assayed the time-course of GABA expression and found that it was first visible at about 24 hpf, shortly after the time at which *met *is expressed in PMNs. Because the GABA antibody cross-reacts with an antigen in the muscle (see Figure 8a,b), we confirmed PMN expression of GABA using a riboprobe to *gad1*, the synthetic enzyme for GABA [48,82] (Figure 8d–e"). Expression of *gad1 *and GABA in PMNs of *met *MO-injected embryos is in stark contrast to control embryos, in which PMNs never express GABA (Figure 8a–e"; see also [81]). To confirm that expression of GABA in CaPs required Met function, we coinjected *mMet *mRNA with *met *translation blocking MOs (Figure 8f–f") and found that this abolished GABA expression in CaPs, whereas embryos coinjected with *lacZ *mRNA and *met *MOs expressed GABA in CaPs (Figure 8g–g"). These results show that not only do CaPs lacking Met function have a motoneuron/interneuron hybrid axonal morphology, they also express both motoneuron and interneuron neurotransmitters.

**Table 5:** CaPs express GABA in embryos in which Met is knocked down

	Number of embryos	Percent of GABA^+ ^CaPs
Control	135	0
*met *MO	88	70
U0126	56	85
SB203580	88	0

CaPs were analyzed in four segments of each embryo. In all cases, CaPs in all four segments had the same GABA phenotype; they were either all GABA^+ ^or all GABA^-^.

### GABA expression in CaP may be regulated by the MEK1/2 but not by the p38 or Akt pathways

**Figure 9:**
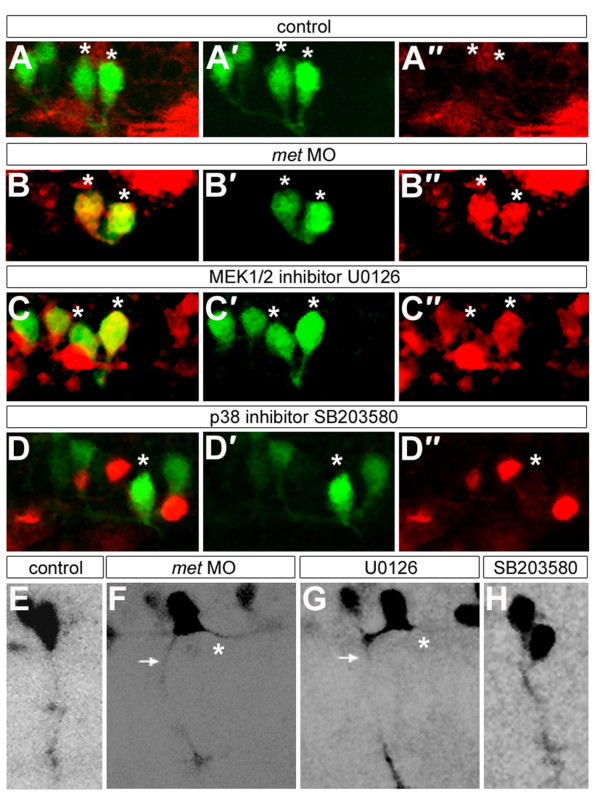
**Met signaling may influence CaP axonal and neurotransmitter phenotypes through the MEK1/2 pathway**. All panels show *mn2Et *transgenic embryos. **(a-d") **Embryos at 26 hpf showing GFP (green) and GABA (red); asterisks indicate CaPs or CaP/VaP pairs. (a',b',c',d') Only the green channel is shown; (a",b",c",d") only the red channel of the micrographs shown in (a,b,c,d) is shown. CaPs express GABA in U0126-treated embryos (c-c") and *met *MO-injected embryos (b-b") but not in controls (a-a") or in SB203580-treated embryos (d-d"). **(e-h) **Some CaPs have both peripheral (arrows) and central (asterisks) axons in U0126-treated embryos (g) and *met *MO-injected embryos (f); CaPs have only have peripheral axons in controls (e) and in SB203580-treated embryos (h). Scale bars, 10 μm.

We used pharmacological inhibitors to learn which Met-activated intracellular signaling pathway(s) transduced the signal required to prevent GABA expression in CaP. Embryos were exposed to inhibitors of these pathways from 16–26 hpf, then fixed and examined for expression of GABA in CaPs (Table 5). Exposure to the MEK1/2 signaling inhibitor U0126 resulted in GABA-positive CaPs, similar to results observed in *met *MO-injected embryos (Figure 9a–c"). In addition, some CaPs in U0126-treated embryos had both peripheral and central axons, similar to *met *MO-injected embryos (Table 3; Figure 9e–g). Together these results suggest that the CaP axonal and neurotransmitter phenotypes seen in the absence of Met function are likely to result from lack of Met activation of MEK1/2. We also treated embryos with SB203580, which inhibits the Akt and p38 pathways and blocked SMN development. In contrast to the effect of U0126, CaPs in embryos exposed to SB203580 had the same neurotransmitter (Figure 9d–d"; Table 5) and axonal (Figure 9h; Table 3) phenotype as control CaPs, suggesting that signaling via p38 and Akt is not involved in regulating CaP neurotransmitter and axon phenotype.

## Discussion

We report two key findings about the role of the Met receptor tyrosine kinase in motoneuron development. First, Met is required for formation of some zebrafish SMNs. Our experiments suggest that this role of Met acts through the p38 and/or Akt signaling cascade. Second, Met is required to prevent CaP motoneurons from co-expressing features of motoneurons and interneurons, including axon pathway and neurotransmitter phenotype. Our experiments suggest that this role of Met acts through the MEK1/2 signaling cascade. We discuss each of these observations in turn.

### Met is necessary for formation of some zebrafish secondary motoneurons

In chick, mouse, and rat, *Met *is expressed in a subset of spinal motoneurons and the Met ligand HGF is important for the differentiation of these cells [14,16,17,19]. *Met *appears to be expressed primarily in limb-innervating lateral motor column motoneurons and HGF acts as a chemoattractant for the axons of these cells *in vitro *and *in vivo*, as well as promoting their survival through the period of normal programmed cell death when tested *in vitro *[16,17,19].

In zebrafish, *met *is also expressed in a subset of spinal motoneurons, in this case in all primary and at least some secondary motoneurons of the medial motor column. In the case of SMNs, Met appears to be required for formation of these cells, as their number is significantly reduced when Met activity is knocked down; whether or not this is the case for the HGF-dependent limb-innervating motoneurons of chick, mouse, or rat has not been reported. The decrease in SMN number when Met is knocked down might result from death of SMNs or their progenitors. We did not see increased cell death, suggesting an alternative possibility that SMNs differentiated as interneurons, as we have previously seen in the absence of Islet1 [81] or Nkx6 proteins [65,83]. Consistent with this possibility, at 48 hpf there were more interneuron-like axons within the spinal cord and somata in positions consistent with spinal interneurons in *Tg(gata2:GFP) *embryos injected with *met *MOs than in *Tg(gata2:GFP) *control embryos.

To begin to learn which intracellular signaling pathways may be involved in Met-mediated SMN formation, we exposed embryos to inhibitors that act on specific pathways downstream of Met activation. Our results suggest that the p38 and/or Akt pathways are required for normal development of SMNs. However, a caveat to this interpretation is that the phenotype was much more severe when these pathways were blocked than when Met was knocked down using MOs. One possible explanation is that Met was incompletely knocked down by our MOs but completely blocked by the pharmacological inhibitor. Alternatively, because these pathways are activated by other receptors in addition to Met, pharmacological blockade may lead to more widespread effects, including effects that are non cell-autonomous (see below). In the future it will be important to learn which other receptors activate these pathways in SMNs and how this is related to activation of these pathways by Met. Finally, the expression of both zebrafish *p38 *genes is widespread throughout early development [79], raising the possibility that p38 activated pathways could have both cell-autonomous and non cell-autonomous effects on SMN development.

### Met prevents primary motoneurons from expressing interneuron-like properties

We have previously found that knocking down function of several transcription factors expressed in PMNs, and in some cases in their progenitors, results in these cells expressing interneuron-like properties. Thus, in the absence of Islet1, PMNs develop interneuron-like axons within the spinal cord, rather than peripheral axons, and many of these cells express the interneuron neurotransmitter GABA [81]. Similarly, in the absence of Nkx6 transcription factors, MiPs develop a hybrid phenotype in which they have both peripheral axons that innervate muscle and central axons that extend within the spinal cord, although these cells do not express GABA [83]. Here we report that knocking down Met function causes CaPs to express a hybrid phenotype in which many of them have both a peripheral axon innervating muscle and a central axon extending within the spinal cord. In addition, these cells co-express cholinergic and GABAergic properties. Despite expression of GABA, PMNs are still able to activate muscle in the absence of Met function. However, the touch response is significantly slower than in control embryos at developmental stages at which this behavior is likely to be mediated primarily by PMNs. Thus, this slower response is probably a result of impairment in PMN function.

The ability of PMNs to develop a hybrid phenotype in the absence of Met reveals a degree of plasticity not previously reported for motoneurons. Previous studies showed that the absence of specific transcription factors in motoneuron progenitors [9,84-89] or in newly post-mitotic motoneurons [83] allows these cells to co-express motoneuron and interneuron properties. These studies reveal the importance of specific transcription factors in preventing motoneurons from developing interneuron properties early in their development. Other studies have shown that postmitotic motoneurons can change their identity from one motoneuron subtype to another in response to environmental cues [90] and that environmental signals can override genetic programs and cause motor axons to extend along aberrant pathways [91]. These studies reveal plasticity in motoneuron subtype specification. In contrast, here we show that motoneurons are able to express interneuron-like properties at late stages of development. Zebrafish *met *is expressed in PMNs about 6–8 hours after they initially extend growth cones and after their axons have extended to and innervated their specific target muscles. Thus, our studies raise the question of whether the ability to develop interneuron characteristics long after their peripheral axons innervate their muscle targets is a general feature of motoneurons. It is well-known that at least some neural crest-derived peripheral neurons have long-lived phenotypic plasticity [10,11], but it is not typically believed that this is the case for central neurons. This issue is particularly important because a recent study has shown that forcing motoneurons to release neurotransmitters other than ACh causes their muscle targets to express receptors to the motoneuron-expressed neurotransmitters [7], potentially leading to inappropriate muscle responses to motoneuron activation.

The late expression of *met *in PMNs raises the interesting question of what prevents CaPs from extending interneuron-like axons and expressing GABA at stages prior to *met *expression. Although we do not have an answer to this question, we hypothesize that many different factors are required to prevent motoneurons from expressing interneuron-like properties. Consistent with this hypothesis, several transcription factors, including Islet1 [81], Nkx6 [85,88,65,83], Lhx3 [9], Hb9 [9] and AML1/Runx1 [89] have been shown to prevent various types of motoneurons from adopting interneuron-like properties. It is not yet clear, but will be exciting to learn, the identities of the downstream targets of these transcription factors and how they regulate different aspects of interneuron development. We predict that different downstream targets prevent motoneurons from expressing different interneuron properties at different developmental stages. We have proposed that zebrafish PMNs have a high propensity to develop into motoneuron/interneuron hybrids because, as has been postulated from studies in mammals [86], zebrafish motoneurons are closely-related to specific types of interneurons [83]. In zebrafish and chick, both motoneurons and interneurons can arise from a single progenitor [92-94]; whether this is also the case in mammals is unknown because single progenitor labeling experiments have not been reported.

### GABA expression in developing motoneurons

Although vertebrate motoneurons are generally considered exclusively cholinergic, several recent studies provide evidence that mammalian spinal motoneurons can release both ACh and glutamate at central synapses on Renshaw cells [4-6]. However, ACh is still thought to be the only neurotransmitter that mediates motoneuron activation of skeletal muscle [4-6]. Thus, it is surprising that during early development, frog muscles express not only AChRs at the nascent neuromuscular junction (NMJ) but also several other types of neurotransmitter receptors, including glutamate receptors, glycine receptors and GABA receptors [7]. From experiments in which they altered motoneuronal neurotransmitter expression, Borodinsky and Spitzer [7] have argued that the final complement of receptors at the NMJ results from matching the neurotransmitter released by motoneurons with the receptors on muscle cells. In their studies, they never saw GABA expression by motoneurons under control conditions. However, several earlier studies reported transient expression of GABA in motoneurons in chick, monkey [95] and rat [96]. GABA may act not only as a neurotransmitter, but also as a trophic factor during development [97,98], and it may be important for integrating developing neurons into circuits [99]. These features might explain early transient expression in neurons that do not normally use GABA as a neurotransmitter. However, neither we nor others have reported GABA expression in zebrafish spinal motoneurons, and the issue of whether transient GABA expression is a common feature of vertebrate spinal motoneurons remains unresolved.

## Conclusion

It has been known for many years that environmental signals can alter subtype specification in newly post-mitotic motoneurons [90]. Here we show that motoneurons retain the ability to develop interneuron-like characteristics, including both axon trajectory and neurotransmitter phenotype, long after they have innervated their muscle targets. In zebrafish, motoneurons and some types of interneurons are generated from the same progenitor domain [92-94], and previous studies showed that in the absence of Notch signaling motoneurons are the preferred fate of cells within that domain [94,100]. Here we suggest that despite this, motoneurons may require continuous signaling to prevent them from developing interneuron-like properties. Our current results also show that motoneurons that co-express interneuron-like properties can still innervate target muscle. In addition, we suggest that the Met receptor tyrosine kinase acts through different intracellular signaling cascades to affect distinct aspects of development in different motoneuron subtypes.

## Abbreviations

ACh: Acetylcholine; AChR: ACh receptor; áBTX: ábungarotoxin; ChAT: Choline acetyltransferase; GABA: Gamma-amino butyric acid; GFP: Green fluorescent protein; HGF: Hepatocyte growth factor; MAPK: Mitogen activated protein kinase; MO: Morpholino antisense oligonucleotide; NMJ: neuromuscular junction; PI3K: Phosphatidylinositol 3-kinase; PMN: Primary motoneuron; SMN: Secondary motoneuron.

## Competing interests

The authors declare that they have no competing interests.

## Authors' contributions

AT carried out all of the experiments described in this paper and helped draft the manuscript. JSE participated in the conception and design of the study and helped draft the manuscript. All authors read and approved the final manuscript.

## References

[B1] Marder E, Eisen JS (1984). Transmitter identification of pyloric neurons: electrically coupled neurons use different transmitters. J Neurophysiol.

[B2] Clarac F, Pearlstein E (2007). Invertebrate preparations and their contribution to neurobiology in the second half of the 20th century. Brain Res Rev.

[B3] Eisen JS (1998). Genetic and molecular analyses of motoneuron development. Curr Opin Neurobiol.

[B4] Herzog E, Landry M, Buhler E, Bouali-Benazzouz R, Legay C, Henderson CE, Nagy F, Dreyfus P, Giros B, El Mestikawy S (2004). Expression of vesicular glutamate transporters, VGLUT1 and VGLUT2, in cholinergic spinal motoneurons. Eur J Neurosci.

[B5] Nishimaru H, Restrepo CE, Ryge J, Yanagawa Y, Kiehn O (2005). Mammalian motor neurons corelease glutamate and acetylcholine at central synapses. Proc Natl Acad Sci U S A.

[B6] Mentis GZ, Alvarez FJ, Bonnot A, Richards DS, Gonzalez-Forero D, Zerda R, O'Donovan MJ (2005). Noncholinergic excitatory actions of motoneurons in the neonatal mammalian spinal cord. Proc Natl Acad Sci U S A.

[B7] Borodinsky LN, Spitzer NC (2007). Activity-dependent neurotransmitter-receptor matching at the neuromuscular junction. Proc Natl Acad Sci U S A.

[B8] Tanabe Y, William C, Jessell TM (1998). Specification of motor neuron identity by the MNR2 homeodomain protein. Cell.

[B9] Thaler JP, Lee SK, Jurata LW, Gill GN, Pfaff SL (2002). LIM factor Lhx3 contributes to the specification of motor neuron and interneuron identity through cell-type-specific protein-protein interactions. Cell.

[B10] Black IB, Patterson PH (1980). Developmental regulation of neurotransmitter phenotype. Curr Top Dev Biol.

[B11] Landis SC (1990). Target regulation of neurotransmitter phenotype. Trends Neurosci.

[B12] Borodinsky LN, Root CM, Cronin JA, Sann SB, Gu X, Spitzer NC (2004). Activity-dependent homeostatic specification of transmitter expression in embryonic neurons. Nature.

[B13] Okura Y, Arimoto H, Tanuma N, Matsumoto K, Nakamura T, Yamashima T, Miyazawa T, Matsumoto Y (1999). Analysis of neurotrophic effects of hepatocyte growth factor in the adult hypoglossal nerve axotomy model. Eur J Neurosci.

[B14] Wong V, Glass DJ, Arriaga R, Yancopoulos GD, Lindsay RM, Conn G (1997). Hepatocyte growth factor promotes motor neuron survival and synergizes with ciliary neurotrophic factor. J Biol Chem.

[B15] Birchmeier C, Birchmeier W, Gherardi E, Vande Woude GF (2003). Met, metastasis, motility and more. Nat Rev Mol Cell Biol.

[B16] Ebens A, Brose K, Leonardo ED, Hanson MG, Bladt F, Birchmeier C, Barres BA, Tessier-Lavigne M (1996). Hepatocyte growth factor/scatter factor is an axonal chemoattractant and a neurotrophic factor for spinal motor neurons. Neuron.

[B17] Yamamoto Y, Livet J, Pollock RA, Garces A, Arce V, deLapeyriere O, Henderson CE (1997). Hepatocyte growth factor (HGF/SF) is a muscle-derived survival factor for a subpopulation of embryonic motoneurons. Development.

[B18] Caton A, Hacker A, Naeem A, Livet J, Maina F, Bladt F, Klein R, Birchmeier C, Guthrie S (2000). The branchial arches and HGF are growth-promoting and chemoattractant for cranial motor axons. Development.

[B19] Novak KD, Prevette D, Wang S, Gould TW, Oppenheim RW (2000). Hepatocyte growth factor/scatter factor is a neurotrophic survival factor for lumbar but not for other somatic motoneurons in the chick embryo. J Neurosci.

[B20] Sun W, Funakoshi H, Nakamura T (2002). Localization and functional role of hepatocyte growth factor (HGF) and its receptor c-met in the rat developing cerebral cortex. Brain Res Mol Brain Res.

[B21] Hanson MG, Landmesser LT (2003). Characterization of the circuits that generate spontaneous episodes of activity in the early embryonic mouse spinal cord. J Neurosci.

[B22] Hayashi Y, Kawazoe Y, Sakamoto T, Ojima M, Wang W, Takazawa T, Miyazawa D, Ohya W, Funakoshi H, Nakamura T, Watabe K (2006). Adenoviral gene transfer of hepatocyte growth factor prevents death of injured adult motoneurons after peripheral nerve avulsion. Brain Res.

[B23] Helmbacher F, Dessaud E, Arber S, deLapeyriere O, Henderson CE, Klein R, Maina F (2003). Met signaling is required for recruitment of motor neurons to PEA3-positive motor pools. Neuron.

[B24] Segarra J, Balenci L, Drenth T, Maina F, Lamballe F (2006). Combined Signaling through ERK, PI3K/AKT, and RAC1/p38 Is Required for Met-triggered Cortical Neuron Migration. J Biol Chem.

[B25] Xiao GH, Jeffers M, Bellacosa A, Mitsuuchi Y, Vande Woude GF, Testa JR (2001). Anti-apoptotic signaling by hepatocyte growth factor/Met via the phosphatidylinositol 3-kinase/Akt and mitogen-activated protein kinase pathways. Proc Natl Acad Sci U S A.

[B26] Zimmermann S, Moelling K (1999). Phosphorylation and regulation of Raf by Akt (protein kinase B). Science.

[B27] Rane MJ, Coxon PY, Powell DW, Webster R, Klein JB, Pierce W, Ping P, McLeish KR (2001). p38 Kinase-dependent MAPKAPK-2 activation functions as 3-phosphoinositide-dependent kinase-2 for Akt in human neutrophils. J Biol Chem.

[B28] Westermarck J, Li SP, Kallunki T, Han J, Kahari VM (2001). p38 mitogen-activated protein kinase-dependent activation of protein phosphatases 1 and 2A inhibits MEK1 and MEK2 activity and collagenase 1 (MMP-1) gene expression. Mol Cell Biol.

[B29] Rivas MA, Carnevale RP, Proietti CJ, Rosemblit C, Beguelin W, Salatino M, Charreau EH, Frahm I, Sapia S, Brouckaert P, Elizalde PV, Schillaci R (2007). TNFalpha acting on TNFR1 promotes breast cancer growth via p42/P44 MAPK, JNK, Akt and NF-kappaB-dependent pathways. Exp Cell Res.

[B30] Cui H, Cai F, Belsham DD (2006). Leptin signaling in neurotensin neurons involves STAT, MAP kinases ERK1/2, and p38 through c-Fos and ATF1. Faseb J.

[B31] Nelson JM, Fry DW (2001). Akt, MAPK (Erk1/2), and p38 act in concert to promote apoptosis in response to ErbB receptor family inhibition. J Biol Chem.

[B32] Porter AC, Vaillancourt RR (1998). Tyrosine kinase receptor-activated signal transduction pathways which lead to oncogenesis. Oncogene.

[B33] Chen JH, Liu TY, Wu CW, Chi CW (2001). Nonsteroidal anti-inflammatory drugs for treatment of advanced gastric cancer: cyclooxygenase-2 is involved in hepatocyte growth factor mediated tumor development and progression. Med Hypotheses.

[B34] Vlahos CJ, Matter WF, Hui KY, Brown RF (1994). A specific inhibitor of phosphatidylinositol 3-kinase, 2-(4-morpholinyl)-8-phenyl-4H-1-benzopyran-4-one (LY294002). J Biol Chem.

[B35] Favata MF, Horiuchi KY, Manos EJ, Daulerio AJ, Stradley DA, Feeser WS, Van Dyk DE, Pitts WJ, Earl RA, Hobbs F, Copeland RA, Magolda RL, Scherle PA, Trzaskos JM (1998). Identification of a novel inhibitor of mitogen-activated protein kinase kinase. J Biol Chem.

[B36] Lali FV, Hunt AE, Turner SJ, Foxwell BM (2000). The pyridinyl imidazole inhibitor SB203580 blocks phosphoinositide-dependent protein kinase activity, protein kinase B phosphorylation, and retinoblastoma hyperphosphorylation in interleukin-2-stimulated T cells independently of p38 mitogen-activated protein kinase. J Biol Chem.

[B37] Cuenda A, Rouse J, Doza YN, Meier R, Cohen P, Gallagher TF, Young PR, Lee JC (1995). SB 203580 is a specific inhibitor of a MAP kinase homologue which is stimulated by cellular stresses and interleukin-1. FEBS Lett.

[B38] Lewis KE, Eisen JS (2003). From cells to circuits: development of the zebrafish spinal cord. Prog Neurobiol.

[B39] Eisen JS, Myers PZ, Westerfield M (1986). Pathway selection by growth cones of identified motoneurones in live zebra fish embryos. Nature.

[B40] Eisen JS, Pike SH, Romancier B (1990). An identified motoneuron with variable fates in embryonic zebrafish. Journal of Neuroscience.

[B41] Myers PZ, Eisen JS, Westerfield M (1986). Development and axonal outgrowth of identified motoneurons in the zebrafish. Journal of Neuroscience.

[B42] Pike SH, Melancon EF, Eisen JS (1992). Pathfinding by zebrafish motoneurons in the absence of normal pioneer axons. Development.

[B43] Myers PZ (1985). Spinal motoneurons of the larval zebrafish. J Comp Neurol.

[B44] Westerfield M, McMurray JV, Eisen JS (1986). Identified motoneurons and their innervation of axial muscles in the zebrafish. Journal of Neuroscience.

[B45] Uemura O, Okada Y, Ando H, Guedj M, Higashijima S, Shimazaki T, Chino N, Okano H, Okamoto H (2005). Comparative functional genomics revealed conservation and diversification of three enhancers of the isl1 gene for motor and sensory neuron-specific expression. Dev Biol.

[B46] McWhorter ML, Monani UR, Burghes AH, Beattie CE (2003). Knockdown of the survival motor neuron (Smn) protein in zebrafish causes defects in motor axon outgrowth and pathfinding. J Cell Biol.

[B47] Pineda RH, Svoboda KR, Wright MA, Taylor AD, Novak AE, Gamse JT, Eisen JS, Ribera AB (2006). Knockdown of Nav1.6a Na+ channels affects zebrafish motoneuron development. Development.

[B48] Higashijima S, Mandel G, Fetcho JR (2004). Distribution of prospective glutamatergic, glycinergic, and GABAergic neurons in embryonic and larval zebrafish. J Comp Neurol.

[B49] Higashijima S, Schaefer M, Fetcho JR (2004). Neurotransmitter properties of spinal interneurons in embryonic and larval zebrafish. J Comp Neurol.

[B50] Bernhardt RR, Patel CK, Wilson SW, Kuwada JY (1992). Axonal trajectories and distribution of GABAergic spinal neurons in wildtype and mutant zebrafish lacking floor plate cells. J Comp Neurol.

[B51] Segawa H, Miyashita T, Hirate Y, Higashijima S, Chino N, Uyemura K, Kikuchi Y, Okamoto H (2001). Functional repression of Islet-2 by disruption of complex with Ldb impairs peripheral axonal outgrowth in embryonic zebrafish. Neuron.

[B52] Balciunas D, Davidson AE, Sivasubbu S, Hermanson SB, Welle Z, Ekker SC (2004). Enhancer trapping in zebrafish using the Sleeping Beauty transposon. BMC Genomics.

[B53] Meng A, Tang H, Ong BA, Farrell MJ, Lin S (1997). Promoter analysis in living zebrafish embryos identifies a cis-acting motif required for neuronal expression of GATA-2. Proc Natl Acad Sci U S A.

[B54] Picker A, Scholpp S, Bohli H, Takeda H, Brand M (2002). A novel positive transcriptional feedback loop in midbrain-hindbrain boundary development is revealed through analysis of the zebrafish pax2.1 promoter in transgenic lines. Development.

[B55] Kimmel CB, Ballard WW, Kimmel SR, Ullmann B, Schilling TF (1995). Stages of embryonic development of the zebrafish. Dev Dyn.

[B56] Haines L, Neyt C, Gautier P, Keenan DG, Bryson-Richardson RJ, Hollway GE, Cole NJ, Currie PD (2004). Met and Hgf signaling controls hypaxial muscle and lateral line development in the zebrafish. Development.

[B57] Ma PC, Tretiakova MS, Nallasura V, Jagadeeswaran R, Husain AN, Salgia R (2007). Downstream signalling and specific inhibition of c-MET/HGF pathway in small cell lung cancer: implications for tumour invasion. Br J Cancer.

[B58] Maina F, Pante G, Helmbacher F, Andres R, Porthin A, Davies AM, Ponzetto C, Klein R (2001). Coupling Met to specific pathways results in distinct developmental outcomes. Mol Cell.

[B59] Hauptmann G, Gerster T (1994). Two-color whole-mount in situ hybridization to vertebrate and Drosophila embryos. Trends Genet.

[B60] Appel B, Korzh V, Glasgow E, Thor S, Edlund T, Dawid IB, Eisen JS (1995). Motoneuron fate specification revealed by patterned LIM homeobox gene expression in embryonic zebrafish. Development.

[B61] Crow MT, Stockdale FE (1986). Myosin expression and specialization among the earliest muscle fibers of the developing avian limb. Dev Biol.

[B62] Patel NH, Martin-Blanco E, Coleman KG, Poole SJ, Ellis MC, Kornberg TB, Goodman CS (1989). Expression of engrailed proteins in arthropods, annelids, and chordates. Cell.

[B63] Downes GB, Granato M (2004). Acetylcholinesterase function is dispensable for sensory neurite growth but is critical for neuromuscular synapse stability. Dev Biol.

[B64] Williams JA, Holder N (2000). Cell turnover in neuromasts of zebrafish larvae. Hear Res.

[B65] Cheesman SE, Layden MJ, Von Ohlen T, Doe CQ, Eisen JS (2004). Zebrafish and fly Nkx6 proteins have similar CNS expression patterns and regulate motoneuron formation. Development.

[B66] Zeller J, Schneider V, Malayaman S, Higashijima S, Okamoto H, Gui J, Lin S, Granato M (2002). Migration of zebrafish spinal motor nerves into the periphery requires multiple myotome-derived cues. Dev Biol.

[B67] Carrel TL, McWhorter ML, Workman E, Zhang H, Wolstencroft EC, Lorson C, Bassell GJ, Burghes AH, Beattie CE (2006). Survival motor neuron function in motor axons is independent of functions required for small nuclear ribonucleoprotein biogenesis. J Neurosci.

[B68] Beattie CE, Carrel TL, McWhorter ML (2007). Fishing for a mechanism: using zebrafish to understand spinal muscular atrophy. J Child Neurol.

[B69] Melancon E, Liu DW, Westerfield M, Eisen JS (1997). Pathfinding by identified zebrafish motoneurons in the absence of muscle pioneers. Journal of Neuroscience.

[B70] Saint-Amant L, Drapeau P (1998). Time course of the development of motor behaviors in the zebrafish embryo. J Neurobiol.

[B71] Brustein E, Saint-Amant L, Buss RR, Chong M, McDearmid JR, Drapeau P (2003). Steps during the development of the zebrafish locomotor network. J Physiol Paris.

[B72] Trevarrow B, Marks DL, Kimmel CB (1990). Organization of hindbrain segments in the zebrafish embryo. Neuron.

[B73] Hatta K, Bremiller R, Westerfield M, Kimmel CB (1991). Diversity of expression of engrailed-like antigens in zebrafish. Development.

[B74] Devoto SH, Melancon E, Eisen JS, Westerfield M (1996). Identification of separate slow and fast muscle precursor cells in vivo, prior to somite formation. Development.

[B75] Liu DW, Westerfield M (1992). Clustering of muscle acetylcholine receptors requires motoneurons in live embryos, but not in cell culture. J Neurosci.

[B76] Beattie CE, Melancon E, Eisen JS (2000). Mutations in the stumpy gene reveal intermediate targets for zebrafish motor axons. Development.

[B77] Fashena D, Westerfield M (1999). Secondary motoneuron axons localize DM-GRASP on their fasciculated segments. J Comp Neurol.

[B78] Robu ME, Larson JD, Nasevicius A, Beiraghi S, Brenner C, Farber SA, Ekker SC (2007). p53 activation by knockdown technologies. PLoS Genet.

[B79] Krens SF, He S, Spaink HP, Snaar-Jagalska BE (2006). Characterization and expression patterns of the MAPK family in zebrafish. Gene Expr Patterns.

[B80] Moon SJ, Fujikawa Y, Nishihara T, Kono S, Kozono K, Ikenaga T, Esaka M, Iijima N, Nagamatsu Y, Yoshida M, Uematsu K (2005). Partial cloning and expression of mRNA coding choline acetyltransferase in the spinal cord of the goldfish, Carassius auratus. Comp Biochem Physiol B Biochem Mol Biol.

[B81] Hutchinson SA, Eisen JS (2006). Islet1 and Islet2 have equivalent abilities to promote motoneuron formation and to specify motoneuron subtype identity. Development.

[B82] Martin SC, Heinrich G, Sandell JH (1998). Sequence and expression of glutamic acid decarboxylase isoforms in the developing zebrafish. J Comp Neurol.

[B83] Hutchinson SA, Cheesman SE, Hale LA, Boone JQ, Eisen JS (2007). Nkx6 proteins specify one zebrafish primary motoneuron subtype by regulating late islet1 expression. Development.

[B84] Arber S, Han B, Mendelsohn M, Smith M, Jessell TM, Sockanathan S (1999). Requirement for the homeobox gene Hb9 in the consolidation of motor neuron identity. Neuron.

[B85] Sander M, Paydar S, Ericson J, Briscoe J, Berber E, German M, Jessell TM, Rubenstein JL (2000). Ventral neural patterning by Nkx homeobox genes: Nkx6.1 controls somatic motor neuron and ventral interneuron fates. Genes Dev.

[B86] Shirasaki R, Pfaff SL (2002). Transcriptional codes and the control of neuronal identity. Annu Rev Neurosci.

[B87] Thaler J, Harrison K, Sharma K, Lettieri K, Kehrl J, Pfaff SL (1999). Active suppression of interneuron programs within developing motor neurons revealed by analysis of homeodomain factor HB9. Neuron.

[B88] Vallstedt A, Muhr J, Pattyn A, Pierani A, Mendelsohn M, Sander M, Jessell TM, Ericson J (2001). Different levels of repressor activity assign redundant and specific roles to Nkx6 genes in motor neuron and interneuron specification. Neuron.

[B89] Stifani N, Freitas AR, Liakhovitskaia A, Medvinsky A, Kania A, Stifani S (2008). Suppression of interneuron programs and maintenance of selected spinal motor neuron fates by the transcription factor AML1/Runx1. Proc Natl Acad Sci U S A.

[B90] Eisen JS (1991). Determination of primary motoneuron identity in developing zebrafish embryos. Science.

[B91] Sharma K, Leonard AE, Lettieri K, Pfaff SL (2000). Genetic and epigenetic mechanisms contribute to motor neuron pathfinding. Nature.

[B92] Kimmel CB, Warga RM, Kane DA (1994). Cell cycles and clonal strings during formation of the zebrafish central nervous system. Development.

[B93] Park HC, Shin J, Appel B (2004). Spatial and temporal regulation of ventral spinal cord precursor specification by Hedgehog signaling. Development.

[B94] Shin J, Poling J, Park HC, Appel B (2007). Notch signaling regulates neural precursor allocation and binary neuronal fate decisions in zebrafish. Development.

[B95] Philippe E, Gaulin F, Delagrave C, Geffard M (1990). Expression of GABA-immunoreactivity by spinal motoneurons of some vertebrates. Neurosci Lett.

[B96] Ma W, Behar T, Barker JL (1992). Transient expression of GABA immunoreactivity in the developing rat spinal cord. J Comp Neurol.

[B97] Nguyen L, Rigo JM, Rocher V, Belachew S, Malgrange B, Rogister B, Leprince P, Moonen G (2001). Neurotransmitters as early signals for central nervous system development. Cell Tissue Res.

[B98] Owens DF, Kriegstein AR (2002). Developmental neurotransmitters?. Neuron.

[B99] Akerman CJ, Cline HT (2007). Refining the roles of GABAergic signaling during neural circuit formation. Trends Neurosci.

[B100] Appel B, Eisen JS (1998). Regulation of neuronal specification in the zebrafish spinal cord by Delta function. Development.

